# A multilevel analysis of the triple burden of malnutrition in Indonesia: trends and determinants from repeated cross-sectional surveys

**DOI:** 10.1186/s12889-023-16728-y

**Published:** 2023-09-21

**Authors:** Helen Andriani, Erlin Friska, Miftahul Arsyi, Alphyyanto Eko Sutrisno, Alexander Waits, Nurul Dina Rahmawati

**Affiliations:** 1 Department of Health Policy and Administration, Faculty of Public Health, Universitas Indonesia, Depok, Indonesia; 2 Master of Public Health Study Program, Faculty of Public Health, Universitas Indonesia, Depok, Indonesia; 3 Institute of Public Health, International Health Program, National Yang Ming Chiao Tung University, Taipei, Taiwan; 4 Department of Nutrition, Faculty of Public Health, Universitas Indonesia, Depok, Indonesia

**Keywords:** Triple burden, Malnutrition, Mother–child pairs, Indonesia Basic Health Research, Multilevel analysis

## Abstract

**Background:**

Although child malnutrition has been reducing, the coexistence in mothers and children of various forms of malnutrition has continued to rise around the world. In the Indonesian context, a knowledge gap exists on the coexistence of multiple malnutrition burdens. This study examines trends in the coexistence of the triple burden of malnutrition (TBM) among mother–child pairs living in the same house and explores multilevel (individual, household, and community) factors associated with TBM in Indonesia.

**Methods:**

We used data from the 2013 and 2018 Indonesia Basic Health Research, the nationally representative survey of the Indonesian population, as repeated cross-sectional surveys. Study samples were mothers and children (0–59 months old), who resided in the same household and indicated by the same identifier number. The anthropometric measurements of the mothers and children, and the hemoglobin levels of the children were collected. We employed a multilevel mixed-effects model to consider the hierarchical data structure. The model captured the role of cluster, district, provincial differences, and the individual, household, community-level, and TBM status characteristics.

**Results:**

Of 3,891 mother–child pairs analyzed, 24.9% experienced TBM. Girls had 63% higher odds than boys of TBM (aOR: 1.63; 95% CI: 1.30 to 2.03). Significantly lower odds were found in children of mothers who had a gestational age lower than 37 weeks (aOR: 0.72; 95% CI: 0.55 to 0.94). At the household level, children with a father who had a high-school, primary-school, or no school education had significantly higher odds of TBM than children of fathers who had graduated from academy. Children of mothers who visited Antenatal Care (ANC) no more than 6 times had significantly lower odds (aOR: 0.65; 95% CI: 0.47 to 0.88). Children of mothers who consumed Iron and Folic Acid (IFA) supplements had significantly lower odds.

**Conclusion:**

TBM is related to characteristics at not just the individual level but also the family and community levels. To achieve significant outcomes, integrated nutrition interventions in Indonesia should also consider family and community factors.

## Background

Although more children are surviving globally, millions of young children are not developing as they should, reflected by stagnant rates of stunting, wasting, and micronutrient deficiencies, as well as rapidly rising rates of overweight and obesity. The most recent global figures for children under the age of 5 years showed 13 million stunted children, 4.5 million wasted children, and 9.7 million overweight children (i.e., 9% of the global burden of stunting and wasting and 24% of the global burden of overweight) [1]. In addition, 46% of children in Southeast Asia are deficient in micronutrients [1]. Malnutrition has a significant impact on a child’s growth and development, as well as their survival, and is among the leading causes of morbidity and mortality in children around the world [2].

Obesity has long been regarded as a problem in developed countries, but due to rapid changes in food habits and lifestyles, it has recently become a problem in developing countries as well. Despite increasing studies in this field, the literature lacks substantial evidence from Indonesia, one of the world’s most populous developing countries. Indonesia is undergoing rapid economic and epidemiological change [3], which continues to be the leading cause of malnutrition and micronutrient deficiencies in the world. According to the 2018 Indonesia Basic Health Research, 30.8% of children under 5 years of age are still affected by stunting, and 10.2% of children under 5 years of age are still affected by wasting. The prevalence of overweight and obese adults is 35.4% [4]. The most recent Indonesia Nutrition Status Survey (SSGI) data in 2022 showed that 21.6% of children under five years old were stunted, 7.7% were wasted [5]. In 2019, an estimated 38% of children aged 6–59 months were anemic in Indonesia [6]. Among developing countries, Indonesia contributes significantly to child anemia [7].

Multiple factors contribute to the triple burden of malnutrition (TBM), which includes inadequate maternal nutrition; nutrient-deficient diets in infancy and early childhood; and changing food systems that increase exposure to cheap and convenient sugary beverages and unhealthy foods high in salt, sugar, and fat but low in essential nutrients [8]. TBM has also been observed in households where the mother is obese and her children are anemic or undernourished (stunting, underweight, or wasting) [9]. Although many forms of maternal and child malnutrition increasingly coexist [10], few studies have examined TBM. The coexistence of undernutrition and obesity, even within the same household, is often considered paradoxical, although several explanations are possible. People frequently consume inexpensive, unhealthy, and calorie-dense foods as food supplies become scarce; as a result, family members grow simultaneously overweight and undernourished [10].

The immediate causes of child undernutrition are inadequate dietary intake and disease, while indirect causes include household food security, caregiving patterns, inadequate health and environmental sanitation. These underlying issues are caused by a variety of individual, household, and community factors [11]. Many prior studies have stressed the impact of socioeconomic, demographic, household, and environmental factors, parental characteristics, child health and feeding practices, and geographic location on childhood nutrition status [12–15]. The findings of these studies are highly valuable for generating evidence-based or need-based intervention strategies, particularly for developing policy-targeted actions to alleviate TBM in children in Indonesia and other developing countries.

TBM is a relatively new topic of constructive debate, and it has received limited attention from academics. In the developing world, there has been a great deal of research on the double burden of malnutrition [16, 17], including Indonesia [18, 19], but TBM has yet to be thoroughly explored due to a lack of data on the determinants of child and maternal malnutrition. In this study, multilevel modeling improved estimation methodologies by allowing us to analyze individual heterogeneities as well as heterogeneities between clusters. Standard errors of regression coefficients are more trustworthy when clustering in the data is considered carefully [20]. We also assessed trends over time and include maternal non-communicable disease (NCD) factors (diabetes mellitus, hypertension, coronary heart disease, stroke, chronic kidney failure), which have not been widely explored as parental factors in previous studies of malnutrition in Indonesia. This study aimed to examine trends in TBM coexistence in mother–child pairs living in the same house and explore multilevel (individual, household, and community) factors associated with TBM in Indonesia.

## Methods

This study involved a secondary analysis of the last two surveys (2013 and 2018) of the Indonesia Basic Health Research (or Riskesdas), the nationally representative survey of the Indonesian population, analyzed as repeated cross-sectional surveys. The 2013 and 2018 Riskesdas use the same method of data collection, measurement, examination and indicators. The National Institute of Health Research and Development, Ministry of Health, Republic of Indonesia, conducts Riskesdas. Using a two-stage stratified cluster sampling process, the survey sample was selected. For each stage, two sampling frames were employed. Households from all 33 provinces, 497 districts and cities, 11,986 census blocks, 294,959 households, and 1,027,763 household members were interviewed in the 2013 Riskesdas [21]. Interviewed in the 2018 Riskesdas were 282,654 households and 1,017,290 individuals from 29,821 census blocks in all 34 provinces containing the total 514 districts and cities in Indonesia [4]. This study used anthropometric and biochemical indices to evaluate the nutritional status of each child aged 0–59 months. Excluded from the study were mothers with no paired child, missing mothers’ body mass index (BMI) information, and children living elsewhere, resulting the final sample of 1,973 from the 2013 Riskesdas and 1,918 from the 2018 Riskesdas. Both survey versions were analyzed twice to describe the data: separated and mixed. This was the same for bivariate and multivariate analysis.

The anthropometric indices used to assess each child’s nutritional status were calculated using the WHO Multicentre Growth Reference Study Group, 2006 [22]. Children with Z-scores of less than -2 standard deviations for height-for-age (HAZ), weight-for-height (WHZ), and weight-for-age (WAZ) were classified as stunted, wasting, and underweight. The level of hemoglobin in the blood was classified as anemic (< 11 g/dL) or not anemic (≥ 11 g/dL). For mothers aged 15–49 years, we used the WHO BMI classifications of normal (18.5 to 24.99 kg/m2) and overweight or obese (> 25.0 kg/m2) [23]. Overweight or obese mothers and undernourished children (stunting, wasting, or underweight) who were also anemic, was referred to as TBM.

In total, we analyzed 20 potential predictors of TBM, categorized into three main levels: individual- (child characteristics), household- (maternal, paternal, and housing characteristics), and community-level factors. Child characteristics included the child’s age, sex, received vitamin A, breastfeeding status, diarrhea during the last two weeks, weight at birth, and gestational age at birth. Maternal and paternal characteristics included the education status of the mother and father, employment status of the mother and father, maternal age at childbirth, number of antenatal care (ANC) visits, number of iron or folic acid (IFA) supplements used during pregnancy, and maternal NCD status (diabetes mellitus, hypertension, coronary heart disease, stroke, and chronic kidney failure). The housing characteristics included the number of household members, number of children aged under 5 years in the household, and household wealth index. The community factors included the place of residence (urban or rural) and region (Java and Bali, Sumatera, Nusa Tenggara Barat/Nusa Tenggara Timur (NTB/NTT), Kalimantan and Sulawesi, or Maluku and Papua).

Descriptive statistics examined all of the variables that were used. Measures of association between outcome variables and potential predictors were estimated using bivariate analysis. We conducted a multilevel analysis as well as two sequential models with random intercepts. We first built a null model (empty model) to assess the role of the cluster, district, and province without adjusting for region or any other potential predictors at the individual or household level. For each level, the median odds ratio (MOR) measured the association with TBM status. After controlling for each other, Model 1 then investigated the role of cluster, district, and provincial differences, as well as individual- and household-level factors and TBM status. Using a 5% significance level, a backward elimination procedure removed any individual- or household-level parameters not substantially related to the research outcome while cluster, district, and province variables remained in the models. For all predictors maintained in the final model, we obtained all adjusted odds ratios (aORs) and 95% confidence intervals (CIs). Statistical analysis of the data was performed using Stata/MP software and the xtmelogit routine.

## Results

**Fig. 1:**
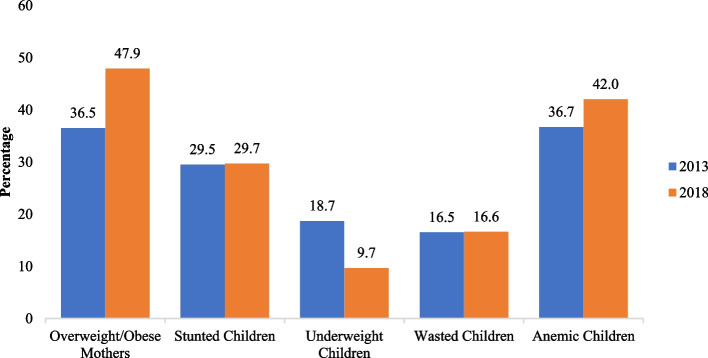
Proportions of nutritional statuses of mothers and children in Indonesia, 2013 and 2018

Figure 1 represents the proportions of the nutritional statuses of mothers and children in Indonesia. Around 47.9% of mothers were overweight or obese. Stunting (29.7%) was the most common form of malnutrition observed, followed by wasting (16.6%) and underweight (9.7%). In addition, around 42.0% of children were anemic.

The characteristics of children and mothers are shown in Table 1. A total of 3,891 children aged 0–59 months were included in the study. Children aged 13–23 months and 24–35 months are mostly observed, at 30% and 31.2%, respectively. The majority of children were boys, received vitamin A, were breastfed, did not experience diarrhea during the last two weeks, and had a normal weight (> 2,500 g) and gestational age (37–40 months) at birth. The most common maternal age at childbirth was 20–29 years (54.5%). We found that the mothers predominantly met the national number of ANC visits recommendation and did not have NCD (diabetes, hypertension, coronary heart disease, stroke, and chronic kidney failure). Most of the fathers and mothers had a primary-school education or were unschooled. Nearly all fathers were employed (96.6%), whereas most mothers were not working (69.2%). The highest household wealth index was Q3 or middle (22.6%). Most of the households had 2–4 family members (52.8%), followed by 5–7 family members (42.8%). More respondents lived in urban than rural areas. The most common regions were Java and Bali (73.1%), which have a dense population, followed by Sumatra, NTB/NTT, and Kalimantan and Sulawesi; the least common was Maluku and Papua at only 0.5%. From 2013 to 2018, children receiving vitamin A increased from 75.0% to 83.8%, a higher proportion of mothers gave birth at older ages, more IFA supplements were used, and more people lived in rural areas (from 39.6% to 45.7%) and Java and Bali (from 68.6% to 77.8%).

**Table 1:** Characteristics of children and mothers in the 2013 and 2018 Indonesia Basic Health Research

Characteristic	2013 (*n* = 1,973)		2018 (*n* = 1,918)		All (*n* = 3,891)	
	n	%	n	%	n	%
***Individual level***						
Child’s age						
0–11 months	96	4.9	316	16.5	412	10.6
12–23 months	734	37.2	432	22.5	1,166	30.0
24–35 months	785	39.8	428	22.3	1,213	31.2
35–47 months	358	18.1	370	19.3	728	18.7
48–59 months	0	0.0	372	19.4	372	9.6
Child’s sex						
Boy	990	50.2	1,019	53.1	2,009	51.6
Girl	983	49.8	899	46.9	1,882	48.4
Received vitamin A						
Yes	1,480	75.0	1,608	83.8	3,088	79.4
No	493	25.0	310	16.2	803	20.6
Breastfed						
Yes	734	37.2	706	36.8	1,440	37.0
No	95	4.8	35	1.8	130	3.3
Do not know	1,143	57.9	1,177	61.4	2,320	59.6
Child had diarrhea during the last 2 weeks						
No	1,741	88.2	1,562	81.4	3,303	84.9
Yes, ≤ 2 weeks ago	120	6.1	230	12.0	350	9.0
Yes, > 2 weeks ago	112	5.7	126	6.6	238	6.1
Weight at birth						
≥ 2,500 g	1,063	53.9	1,128	58.8	2,191	56.3
< 2,500 g	51	2.6	75	3.9	126	3.2
Do not know	859	43.6	715	37.3	1,574	40.5
Gestational age at birth						
37–40 weeks	1,151	58.3	1,290	67.3	2,441	62.7
< 37 weeks	656	33.3	509	26.5	1,165	29.9
> 40 weeks	166	8.4	119	6.2	285	7.3
***Household level***						
Mother’s education						
Academy	147	7.4	137	7.1	284	7.3
High school	529	26.8	578	30.1	1,107	28.5
Middle school	598	30.3	552	28.8	1,150	29.6
Primary school/no school	699	35.5	651	33.9	1,350	34.7
Father’s education						
Academy	168	8.5	154	8.0	322	8.3
High school	577	29.2	624	32.5	1,201	30.9
Middle school	565	28.6	425	22.2	990	25.4
Primary school/no school	663	33.6	715	37.3	1,378	35.4
Mother’s employment						
Not working	1,451	73.5	1,243	64.8	2,694	69.2
Working	522	26.5	675	35.2	1,197	30.8
Father’s employment						
Not working	93	4.7	39	2.0	132	3.4
Working	1,880	95.3	1,879	98.0	3759	96.6
Maternal age at childbirth						
20–29 years	1,298	65.8	822	42.9	2,120	54.5
< 20 years	622	31.6	127	6.6	749	19.2
30–39 years	53	2.7	841	43.8	894	23.0
≥ 40 years	0	0.0	128	6.7	128	3.3
Number of ANC visits						
6 or more	1,374	69.6	1,381	72.0	2,755	70.8
< 6	599	30.4	537	28.0	1,136	29.2
Number of iron/folic acid supplements used during pregnancy						
None	169	8.5	147	7.7	316	8.1
< 90 tablets	715	36.3	820	42.8	1,535	39.4
90 tablets or more	785	39.7	936	48.8	1,721	44.2
Do not remember	305	15.5	15	0.8	320	8.2
Maternal NCD status						
Diabetes mellitus						
No	1,965	99.6	1,915	99.8	3,880	99.7
Yes	8	0.4	3	0.2	11	0.3
Hypertension						
No	1,817	92.1	1798	93,8	3,615	92.9
Yes	156	7.9	119	6,2	275	7.1
Coronary heart disease						
No	1,973	100.0	1,888	98.4	3,861	99.2
Yes	0	0.0	30	1.6	30	0.8
Stroke						
No	1,973	100.0	1,914	99.8	3,887	99.9
Yes	0	0	4	0.2	4	0.1
Chronic kidney failure						
No	1,968	99.8	1,909	99.5	3,877	99.6
Yes	5	0.2	9	0.5	14	0.4
Number of household members						
2–4	1,069	54.2	986	51.4	2,055	52.8
5–7	837	42.4	828	43.2	1,665	42.8
8 +	67	3.4	104	5.4	171	4.4
Number of children aged under 5 years in the household						
1	1,669	84.6	1,708	89.1	3,377	86.8
2	270	13.7	193	10.1	463	11.9
3 +	34	1.7	17	0.9	51	1.3
Household wealth index						
Q5 (richest)	229	11.6	258	13.5	747	19.2
Q4	309	15.7	333	17.4	740	19.0
Q3 (middle)	503	25.5	378	19.7	881	22.6
Q2	517	26.2	431	22.5	850	21.8
Q1 (poorest)	415	21.0	518	27.0	673	17.3
***Community level***						
Residence						
Urban	1,192	60.4	1,041	54.3	2,233	57.4
Rural	781	39.6	877	45.7	1,658	42.6
Region						
Java and Bali	1,352	68.6	1,493	77.8	2,845	73.1
Sumatera	363	18.4	157	8.2	520	13.4
NTB/NTT	72	3.7	127	6.6	199	5.1
Kalimantan and Sulawesi	174	8.8	135	7.0	309	7.9
Maluku and Papua	12	0.6	6	0.3	18	0.5

Table 2 estimates the bivariate associations of individual-, household-, and community-level factors with TBM in mother–child pairs. Across 2013, 2018, and all data, consistently significant associations existed between TBM without adjustment and maternal age at childbirth, ANC visits, mother’s hypertension, and household wealth index. Based on all data, TBM increased when children were not breastfed, children had diarrhea within 2 weeks, fathers were not working, mothers’ childbirth age was under 20 years or over 30 years old, no IFA supplements were taken during pregnancy, mothers were hypertensive, and families lived in an urban setting. For the individual variables, children aged 2.0–2.9 years or 3.0–3.9 years had the highest prevalence of TBM, at 26.1%, compared to children of other ages. A higher burden of TBM existed among girls than boys. Non-breastfed children had a higher risk of TBM (OR: 2.17; 95% CI: 1.49 to 3.16; *p* < 0.001). A higher proportion of children who experienced diarrhea within 2 weeks had a higher risk of TBM (33.8%) than those who did not (24.0%; OR: 1.61; 95% CI: 1.22 to 2.14; *p* < 0.001). For the household variables, working fathers were a significant protector of TBM (OR: 0.61; 95% CI: 0.42 to 0.88; *p* = 0.009). A higher rate of TBM was observed among mothers who were over 40 years of age (39.8%) than among those who were younger (20–29 years; 22.1%; OR: 2.33; 95% CI: 1.61 to 3.37; *p* = 0.001). The lowest TBM was observed for mothers who consumed 90 tablets or more of IFA supplements during pregnancy (OR: 0.79; 95% CI: 0.61 to 1.03; *p* = 0.091). Among mothers with NCDs, TBM was most common (34.0%) with maternal hypertension (OR: 1.61, 95% CI: 1.24 to 2.09; *p* = 0.001). Notably, no mothers had diabetes, coronary heart disease, stroke, or chronic kidney disease. For the community variables, urban mother–child pairs had a higher prevalence of TBM (26.3%) compared to rural mother–child pairs (23.1%).

**Table 2:** Bivariate associations of TBM with characteristics of mother–child pairs in Indonesia

	2013		*P*	2018		*p*	All		*p*
	TBM (*n* = 426)			TBM (*n* = 544)			TBM (*n* = 970)		
	n (%)	OR (95% CI)		n (%)	OR (95% CI)		n (%)	OR (95% CI)	
***Individual level***									
Child’s age									
0–11 months	7 (7.5)	1.00		90 (28.5)	1.00		97 (23.6)	1.00	
12–23 months	140 (19.1)	2.92 (1.34–6.37)	0.007	134 (31.0)	1.13 (0.83–1.54)	0.442	274 (23.5)	0.99 (0.76–1.29)	0.985
24–35 months	186 (23.6)	3.82 (1.75–8.30)	0.001	132 (30.8)	1.12 (0.82–1.53)	0.476	318 (26.1)	1.14 (0.88–1.49)	0.298
35–47 months	93 (26.1)	4.35 (1.97–9.64)	< 0.001	97 (26.2)	0.89 (0.64–1.25)	0.511	190 (26.1)	1.14 (0.86–1.52)	0.338
48–59 months	0 (0.0)	-	-	91 (24.5)	0.81 (0.59–1.13)	0.218	91 (24.9)	1.04 (0.75–1.45)	0.775
Child’s sex									
Boy	186 (18.8)	1.00		286 (28.1)	1.00		472 (23.5)	1.00	
Girl	240 (24.4)	1.40 (1.13–1.73)	0.002	258 (28.7)	1.032 (0.841–1.26)	0.759	498 (26.4)	1.17 (1.01–1.35)	0.032
Received vitamin A									
Yes	307 (20.7)	1.00		461 (28.7)	1.00		768 (24.8)	1.00	
No	120 (24.3)	1.23 (0.97–1.56)	0.093	83 (26.8)	0.91 (0.70–1.19)	0.488	203 (25.2)	1.02 (0.85–1.22)	0.811
Breastfed									
Yes	108 (14.6)	1.00		213 (30.2)	1.00		321 (22.2)	1.00	
No	40 (41.9)	4.22 (2.67–6.65)	< 0.001	10 (28.6)	0.93 (0.43–1.98)	0.842	50 (38.3)	2.17 (1.49–3.16)	< 0.001
Do not know	279 (24.4)	1.88 (1.47–2.40)	< 0.001	321 (27.3)	0.87 (0.71–1.06)	0.170	600 (25.8)	1.21 (1.04–1.42)	0.013
Child had diarrhea during the last 2 weeks									
No	358 (20.6)	1.00		436 (27.9)	1.00		794 (24.0)	1.00	
Yes, ≤ 2 weeks ago	26 (21.5)	1.06 (0.67–1.66)	0.805	70 (30.4)	1.13 (0.84–1.52)	0.415	96 (27.3)	1.19 (0.92–1.52)	0.168
Yes, > 2 weeks ago	43 (38.1)	2.37 (1.59–3.53)	< 0.001	38 (30.2)	1.12 (0.75–1.65)	0.585	81 (33.8)	1.61 (1.22–2.14)	0.001
Weight at birth									
≥ 2,500 g	221 (20.8)			313 (27.7)	1.00		534 (24.3)	1.00	
< 2,500 g	6 (11.2)	0.48 (0.20–1.17)	0.106	20 (26.7)	0.947 (0.55–1.62)	0.841	26 (20.4)	0.79 (0.51–1.24)	0.318
Do not know	200 (23.3)	1.15 (0.93–1.43)	0.190	211 (29.5)	1.090 (0.89–1.34)	0.413	411 (26.1)	1.09 (0.94–1.27)	0.225
Gestational age at birth									
37–40 weeks	265 (23.1)	1.00		359 (27,8)	1.00		624 (25.5)	1.00	
< 37 weeks	111 (17.0)	0.68 (0.53–0.87)	0.002	154 (30.3)	1.13 (0.90–1.41)	0.303	265 (22.7)	0.85 (0.72–1.01)	0.069
> 40 weeks	50 (30.0)	1.42 (0.99–2.04)	0.051	31 (26.1)	0.91 (0.61–1.38)	0.667	81 (28.3)	1.15 (0.87–1.51)	0.314
***Household level***									
Mother’s education									
Academy	56 (38.2)	1.00		32 (23.4)	1.00		88 (31.0)	1.00	
High school	76 (14.3)	0.26 (0.17–0.40)	< 0.001	146 (25.3)	1.11 (0.72–1.70)	0.636	222 (20.0)	0.55 (0.41–0.74)	< 0.001
Middle school	119 (19.9)	0.40 (0.27–0.59)	< 0.001	155 (28.1)	1.28 (0.84–1.96)	0.254	274 (23.8)	0.69 (0.52–0.92)	0.013
Primary school/no school	176 (25.2)	0.54 (0.37–0.79)	0.001	211 (32.4)	1.57 (1.03–2.41)	0.037	387 (28.6)	0.89 (0.67–1.17)	0.423
Father’s education									
Academy	34 (20.4)	1.00		31 (25.0)	1.00		73 (22.7)	1.00	
High school	144 (24.9)	1.29 (0.85–1.97)	0.223	143 (26.6)	1.09 (0.69–1.71)	0.721	310 (25.8)	1.18 (0.88–1.58)	0.249
Middle school	103 (18.3)	0.87 (0.56–1.34)	0.541	105 (28.9)	1.20 (0.77–1.95)	0.401	222 (22.4)	0.98 (0.72–1.32)	0.913
Primary school/no school	145 (21.9)	1.09 (0.72–1.66)	0.664	188 (32.0)	1.41(0.91–2.19)	0.126	364 (26.4)	1.22 (0.91–1.62)	0.173
Mother’s employment									
Not working	301 (20.7)	1.00		366 (29.4)	1.00		667 (24.7)	1.00	
Working	126 (24.1)	1.21 (0.95–1.53)	0.115	178 (26.4)	0.86 (0.70–1.05)	0.144	304 (25.3)	1.03 (0.88–1.20)	0.689
Father’s employment									
Not working	32 (34.2)	1.00		13 (36.1)	1.00		45 (34.6)	1.00	
Working	395 (21.0)	0.51 (0.32–0.79)	0.003	454 (28.8)	0.72 (0.36–1.43)	0.341	924 (24.6)	0.61 (0.42–0.88)	0.009
Maternal age at childbirth									
20–29 years	259 (20.0)	1.00		209 (25.4)	1.00		468 (22.1)	1.00	
< 20 years	153 (24.5)	1.30 (1.03–1.63)	0.024	20 (15.7)	0.55 (0.34–0.90)	0.016	173 (23.0)	1.05 (0.86–1.28)	0.594
30 – 39 years	14 (27.4)	1.50 (0.80–2.80)	0.197	264 (31.4)	1.34 (1.09–1.66)	0.006	278 (31.1)	1.59 (1.33–1.89)	< 0.001
≥ 40 years	0 (0.0)			51 (39.8)	1.94 (1.30–2.91)	0.001	51 (39.8)	2.33 (1.61–3.37)	< 0.001
Number of ANC visits									
6 or more	330 (24.0)	1.00		409 (29.6)	1.00		739 (26.8)	1.00	
< 6	97 (16.1)	0.61 (0.47–0.78)	< 0.001	135 (25.1)	0.80 (0.64–0.99)	0.043	232 (20.4)	0.70 (0.59–0.82)	< 0.001
Number of iron/folic acid supplements used during pregnancy									
None	59 (35.4)	1.00		37 (25.2)	1.00		96 (30.6)	1.00	
< 90 tablets	138 (19.3)	0.43 (0.30–0.63)	< 0.001	233 (28.4)	1.18 (0.79–1.76)	0.415	371 (24.2)	0.72 (0.55–0.94)	0.016
90 tablets or more	177 (22.6)	0.53 (0.37–0.76)	0.001	271 (29.0)	1.21(0.82–1.79)	0.335	448 (26.0)	0.79 (0.61–1.03)	0.091
Do not remember	50 (16.6)	0.36 (0.23–0.56)	< 0.001	3 (20.0)	0.74 (0.24–2.35)	0.613	53 (16.7)	0.45 (0.31–0.66)	< 0.001
Maternal NCD status									
Diabetes mellitus									
No	426 (21.7)	1.00		541 (28.3)	1.00		967 (24.9)	1.00	
Yes	0 (0.0)	-	-	3 (100.0)	-	-	3 (28.1)	1.17 (0.30–4.48)	0.810
Hypertension									
No	382 (21.0)	1.00		382 (28.8)	1.00		876 (24.2)	1.00	
Yes	44 (28.5)	1.50 (1.04–2.16)	0.029	46 (44.7)	2.00 (1.34–2.98)	0.001	93 (34.0)	1.61 (1.24–2.09)	< 0.001
Coronary heart disease									
No	426 (21.6)	1.00		539 (28.5)	1.00		965 (25.0)	1.00	
Yes	0 (0.0)	-	-	5 (16.7)	0.50 (0.19–1.31)	0.158	5 (16.6)	0.59 (0.22–1.57)	0.298
Stroke									
No	426 (21.6)	1.00		543 (28.4)	1.00		969 (25.0)	1.00	
Yes	0 (0.0)	-	-	1 (25.0)	0.84 (0.09–8.12)	0.881	1 (25.0)	1.00 (0.10–9.65)	0.998
Chronic kidney failure									
No	426 (21.6)	1.00		543 (28.4)	1.00		969 (25.0)	1.00	
Yes	0 (0.0)	-	-	1 (11.1)	0.31 (0.04–2.50)	0.274	1 (7.2)	0.23 (0.03–1.78)	0.162
Number of household members									
2–4	250 (23.4)	1.00		277 (28.1)	1.00		527 (25.6)	1.00	
5–7	175 (20.9)	0.86 (0.69–1.07)	0.195	240 (29.0)	1.05 (0.85–1.28)	0.670	415 (25.0)	0.96 (0.82–1.11)	0.611
8 +	1 (1.7)	0.05 (0.01–0.36)	0.002	27 (26.0)	0.90 (0.57–1.43)	0.647	28 (16.5)	0.57 (0.37–0.86)	0.009
Number of children aged under 5 years in the household									
1	385 (23.1)	1.00		482 (28.2)	1.00		867 (25.7)	1.00	
2	40 (15.1)	0.59 (0.41–0.84)	0.003	59 (30.6)	1.12 (0.81–1.56)	0.499	99 (21.5)	0.79 (0.62–1.00)	0.054
3 +	0 (0.0)	-	-	4 (17.6)	0.55 (0.12–2.54)	0.439	4 (5.8)	0.17 (0.05–0.57)	0.004
Household wealth index									
Q5 (richest)	61 (26.8)	1.00		63 (24.4)	1.00		124 (25.5)	1.00	
Q4	66 (21.6)	0.75 (0.50–1.11)	0.157	101 (30.3)	1.41 (1.00–1.98)	0.048	167 (26.1)	1.02 (0.78–1.34)	0.832
Q3 (middle)	70 (14.0)	0.44 (0.30–0.65)	< 0.001	100 (26.5)	1.17 (0.83–1.64)	0.373	170 (19.3)	0.69 (0.53–0.91)	0.008
Q2	152 (29.5)	1.13 (0.80–1.61)	0.464	118 (27.4)	1.11 (0.77–1.60)	0.563	270 (28.5)	1.16 (0.90–1.48)	0.234
Q1 (poorest)	74 (18.0)	0.60 (0.40–0.88)	0.010	162 (31.3)	1.348 (0.93–1.96)	0.117	236 (25.4)	0.99 (0.77–1.27)	0.950
***Community level***									
Residence									
Urban	277 (23.2)	1.00		310 (27.0)	1.00		587 (26.3)	1.00	
Rural	149 (19.0)	0.77 (0.62–0.97)	0.027	234 (26.7)	0.86 (0.70–1.05)	0.137	383 (23.1)	0.84 (0.72–0.97)	0.022
Region									
Java and Bali	279 (20.6)	1.00		440 (29.5)	1.00		719 (25.2)	1.00	
Sumatera	83 (22.9)	1.14 (0.86–1.51)	0.341	22 (14.0)	0.39 (0.24–0.63)	< 0.001	105 (20.2)	0.75 (0.59–0.94)	0.015
NTB/NTT	19 (27.6)	1.46 (0.86–2.49)	0.160	38 (29.9)	1.02 (0.69–1.51)	0.914	57 (29.0)	1.21 (0.88–1.66)	0.235
Kalimantan and Sulawesi	43 (25.0)	1.28 (0.88–1.85)	0.184	41 (30.4)	1.04 (0.72–1.52)	0.824	84 (27.3)	1.11 (0.85–1.44)	0.425
Maluku and Papua	0 (0.0)	-	-	3 (50.0)	2.39 (0.34–17.08)	0.384	3 (17.7)	0.63 (0.18–2.21)	0.478

The results of the multilevel modelling in Table 3 only showed variables that had statistically significant relationships with TBM from the 2013 Riskesdas, 2018 Riskesdas, or both. The null model shows that the MOR of Province in 2013, 2018, and all data was 1.49, 1.34, and 1.22, respectively. The MOR of District had lower values in 2013, 2018, and all data, representing a weaker effect than Province (1.00, 1.35, and 1.15). The stronger effect of Cluster was represented by a higher MOR in 2013, 2018, and all data than Province and District (2.87, 1.37, and 11.82). In 2013, when individual-level factors (gestational age at birth) and household-level factors (father’s employment, number of IFA supplements, and number of household members) were added into the null model (multivariate model), the MORs of District and Cluster changed significantly, whereas the Province MOR changed slightly. The Province MOR decreased by 12%, the District MOR increased by 781%, and the Cluster MOR increased by 722%. The residual heterogeneity between Districts and Clusters (MOR = 8.81 and 23.59) had greater relevance to other variables at each level. In 2018, when household-level (mother’s education, maternal age at childbirth, number of ANC visits, and hypertension) and community-level factors (region) were added to the null model (multivariate model), all MORs changed slightly. The Province MOR decreased by 4%, the District MOR decreased by 6%, and the Cluster MOR decreased by 7%. The residual heterogeneity among Province, District, and Cluster variables was not more relevant than for other variables at each level. In all data, each level’s MOR increased after individual-level factors (sex of child and gestational age at birth) and household-level factors (father’s education, number of ANC visits, and number of IFA supplements) were added into the null model (multivariate model). The Province MOR increased by 26%, the District MOR increased by 33%, and the Cluster MOR increased by 8%. The residual heterogeneity (MOR = 12.89) was more relevant than other variables at the cluster level.

**Table 3:** Multilevel mixed-effects model logistic regression results of TBM

	2013 (*n* = 426)		2018 (*n* = 544)		All (*n* = 970)	
	Multivariate^a^	Null model	Multivariate^b^	Null model	Multivariate^c^	Null model
	aOR (95% CI)	aOR (95% CI)	aOR (95% CI)	aOR (95% CI)	aOR (95% CI)	aOR (95% CI)
***Individual level***						
Child’s sex						
Boy					1.00	
Girl					1.63 (1.30–2.03)***	
Gestational age at birth						
37–40 weeks	1.00				1.00	
< 37 weeks	0.27 (0.15–0.47)***				0.72 (0.55–0.94)*	
> 40 weeks	0.84 (0.26–2.66)				1.18 (0.73–1.88)	
***Household level***						
Mother’s education						
Academy			1.00			
High school			1.19 (0.75–1.89)			
Middle school			1.51 (0.95–2.41)			
Primary school/no school			1.75 (1.11–2.76)*			
Father’s education						
Academy					1.00	
High school					1.78 (1.11–2.84)*	
Middle school					1.25 (0.76–2.05)	
Primary school/no school					1.61 (1.00–2.60)*	
Father’s employment						
Not working	1.00					
Working	0.14 (0.04–0.42)**					
Maternal age at childbirth						
20–29 years			1.00			
< 20 years			0.51 (0.20–0.85)*			
30–39 years			1.34 (1.06–1.68)*			
≥ 40 years			1.94 (1.28–2.96)**			
Number of ANC visits						
6 or more			1.00		1.00	
< 6			0.77 (0.60–0.99)*		0.65 (0.47–0.88)**	
Number of iron/folic acid supplements used during pregnancy						
None	1.00				1.00	
< 90 tablets	0.09 (0.03–0.27)***				0.49 (0.31–0.76)**	
90 tablets or more	0.01 (0.00–0.04)***				0.35 (0.21–0.55)***	
Do not remember	0.02 (0.00–0.08)***				0.14 (0.06–0.31)***	
Maternal NCD status						
Hypertension						
No			1.00			
Yes			1.58 (1.05–2.37)*			
Number of household members						
2–4	1.00					
5–7	1.23 (0.70–2.13)					
8 +	0.07 (0.00–0.11)***					
***Community level***						
Region						
Java and Bali			1.00			
Sumatera			0.37 (0.23–0.61)***			
NTB/NTT			1.05 (0.678–1.62)			
Kalimantan and Sulawesi			1.15 (0.76–1.75)			
Maluku and Papua			2.96 (0.50–17.37)			
Province (MOR)	1.31	1.49	1.28	1.34	1.65	1.22
District (MOR)	8.81	1.00	1.27	1.35	1.71	1.15
Cluster (MOR)	23.59	2.87	1.28	1.37	12.89	11.82

^***^p < 0.001, **p < 0.010, *p < 0.050

^a^Adjusted for gestational age at birth, father’s employment, and number of iron/folic acid supplements

^b^Adjusted for mother’s education, number of ANC visits, hypertension, and region

^c^Adjusted for sex of the child, gestational age at birth, father’s education, number of ANC visits, number of iron/folic acid supplements

In the multivariate model analysis for 2013, we found significantly decreased odds of TBM in children with < 37 weeks gestational age at birth (aOR: 0.27; 95% CI: 0.15 to 0.42). With the father’s employment, the odds of TBM significantly decreased (aOR: 0.14; 95% CI: 0.04 to 0.42). We also found significantly lower odds in mothers who consumed IFA supplements. Additionally, significantly lower odds were found in children from households with 8 or more members (aOR: 0.07; 95% CI: 0.00 to 0.11). In 2018, children of mothers with a primary-school or no education had significantly higher odds of TBM (aOR: 1.75; 95% CI: 1.11 to 2.76). Children of mothers with a maternal age at childbirth outside the range of 20–29 years had significantly higher odds. Children of mothers with at least 6 ANC visits during the pregnancy had significantly lower odds (aOR: 0.77; 95% CI: 0.60 to 0.99). Children of mothers who had hypertension also had significantly higher odds (aOR: 1.58; 95% CI: 1.05 to 2.37). At the community level, only children in Sumatera had lower significant TBM odds (aOR: 0.37; 95% CI: 0.23 to 0.61). In the combined 2013 and 2018 data, girls had 63% higher odds of TBM than boys (aOR: 1.63; 95% CI: 1.30 to 2.03). Significantly lower odds were found in children of mothers with a gestational age lower than < 37 weeks (aOR: 0.72; 95% CI: 0.55 to 0.94). At the household level, children of fathers who had a high-school, primary-school, or no school education had significantly higher odds than those who had graduated from academy (i.e. diploma or higher). Children of mothers who had visited ANC no more than 6 times had significantly lower odds (aOR: 0.65; 95% CI: 0.47 to 0.88). Children of mothers who consumed IFA supplements had significantly lower odds.

## Discussion

This study explored the coexistence trend of TBM in mother–child pairs and the potential predictors related to TBM at multiple levels (individual, household, and community) in Indonesia. This is the first study to use a stratified mixed-effects model and analyze a repeated cross-sectional survey, using nationally representative 2013 and 2018 Indonesia Basic Health Research data. Indonesia is a prime example of TBM. Around one in three children aged under 5 years are stunted, one in 10 children have wasting, and a further 8% are overweight. The prevalence of overweight and obesity has increased from around 36.5% in 2013 to 47.9% in 2018. Maternal overweight or obesity is associated with a nutritional transition situation that contributes to a positive energy balance, meaning higher intake of energy-dense foods and less energy expenditure [9]. The tendency to consume calorie-dense foods with more saturated fat and trans fat with a sedentary lifestyle causes women of childbearing age to gain weight [24].

The prevalence of TBM in Indonesia increased from 21.6% in 2013 to 28.4% in 2018, according to this study. The prevalence of overweight or obese mothers and children’s anemia increased by more than 5%, which contributed to an increase in TBM rates. In 2018, the number of mothers who gave birth when they were older increased. This is one of the main risks for mothers who are overweight or obese, consistent with findings from studies in Myanmar [25] associating obesity with mothers who gave birth at older ages, which increased the prevalence of TBM. The increased prevalence of obesity among mothers in the older age group is a likely explanation for the higher incidence of TBM. As women age, their physical activity and metabolic rate decrease, resulting in a rise in obesity. Additionally, since the energy requirement reduces, even consistent or routine eating may contribute to weight gain in women as they age. Another factor contributing to older women's obesity is that women acquire weight when pregnant and that postpartum weight loss does not always occur [26].

This study showed a unique trend: along with an increase in overweight and obese mothers, we saw an increase in children who were anemic in 2018. This was also discovered by Sunuwar et al. [9] in Nepal, who found a relationship between obesity and iron deficiency in children, contributing to the rise in TBM. The mechanism that could explain TBM has not been thoroughly investigated. A reasonable explanation is that maternal overweight or obesity is a risk factor for child anemia. Obesity and excessive weight gain during pregnancy are associated with an increased risk of low iron serum in neonates. Serum iron and transferrin saturation in cord blood are both lower in obese women than in normal-weight women, resulting in less iron being transferred to the newborn. The overexpression of hepcidin under proinflammatory conditions in overweight and obese mothers results in poor iron transport to the placenta and iron insufficiency in the neonate [27]. After birth, the child keeps growing and needs nourishing food which may not be provided by a regular diet so they become more anemic, stunted, and underweight [28]. Anemia is the most common type of household burden of malnutrition, impacting almost seven out of ten households, according to research in Sub-Saharan Africa [29]. This was also the case in Gambia [30] and Bangladesh [31]; although the malnutrition rate has decreased, the prevalence of anemia in children has not reduced and remains high. This is also probable in Indonesia, as suggested by this study, which indicated an increase in the number of anemic children in 2018, contributing to the rise in TBM. Various individual-, household-, and community-level factors were associated with TBM among mother–child pairs in Indonesia. Future program planning and interventions need to consider a multisectoral approach to reduce TBM.

As a result of unadjusted variables, TBM was found to be higher for non-breastfed children. Additionally, breastfeeding is associated with a decreased risk of stunting [32] and anemia [31] than never breastfeeding. Breastfeeding status had a substantial impact on the anemic status of the mother–child pair. Although breastfeeding functions to transfer nutrients from mother to child, this is challenging when the mother–child pair is anemic. Reduced maternal hemoglobin levels can influence the immunological and nutritional qualities of breast milk at various phases of development. Iron concentrations in breast milk correlate to maternal hemoglobin levels by reducing the levels of serum iron in breast milk [33]. Iron deficiency is the most significant risk factor for anemia in women [30].

IFA supplements used during pregnancy become problematic if they are disregarded. This study found a correlation between the intake of IFA supplements and TBM. Research from 7 South Asian countries showed that IFA tablets can increase newborn health significantly compared to newborn of mothers who consumed no IFA tablets during pregnancy [34]. Alaofè et al. [35] and Eshete et al. [36] also found that a higher risk of anemia and iron deficiency in women and their children was associated with anthropometric deficiencies such as stunting. Therefore, a failure to prevent anemia during pregnancy with IFA supplements will affect the growth and development of children born with conditions such as low birth weight, as well as future chronic malnutrition (wasting, stunting, and underweight) [37]. In our study, younger gestational age was identified as a protective factor against developing TBM. Possible explanation may involve significant association between younger gestational age and low birth weight, along with the accelerated postanal ‘catch-up’ growth in the first 24 months – a common compensatory mechanism for low birth weight [38, 39].

This study indicated that TBM was higher among urban mother–child pairs than in rural areas. The results of research in India were similar. Urban women have a higher prevalence of obesity than their rural counterparts [40]; therefore, TBM is more predominant in urban areas. Urbanization influences lifestyles, consequently increasing the prevalence of obesity among urban women. Insufficient physical activity and sedentary behavior have been identified as risk factors for overweight and obesity in urban women [41]. In addition, Western culture has long competed to create multiple fast-food chains with cheaper prices accessible to everyone, which has influenced the prevalence of obesity in metropolitan regions of Indonesia. Although the raising of maternal obesity in urban areas; cannot be ignored, Eshete et al. [42] observed the highest DBM in rural areas. In addition, significant numbers of children in rural areas still have chronic malnutrition, specifically stunting [43]. TBM is also associated with geographical region. Sumatera island had significant TBM in this study. Sumatera is a developing region in Indonesia that shows the same poor-rich country trend [44]. Moreover, mothers with NCDs such as hypertension showed an association with TBM. This relates to a poor obstetric history that affects the child’s growth during pregnancy until birth [45].

According to the findings, girls are 1.63 times more likely than boys to experience TBM. Studies in Nepal [9] found that boys are more likely than girls to experience TBM. It has been shown that boys are more likely to be malnourished during childhood than girls, and this difference appears as early as the fetal period [46]. After the first six months of life, however, the benefits of exclusive breastfeeding are disproportionate to the higher nutritional deficit in girls. Girls were fed less food than boys. Due to early introduction of foods that boys' growth would falter in the first six months, and girls, whose food consumption is lower, would falter more than boys [47]. Due to the fact that almost 80% of children were above one year old, we estimate that the results of this study put girls at higher risk for TBM.

In 2021, regulation of Indonesian ANC visits increased from 4 to 6 times visits [48]. From 2006 to 2018, the number to complete four ANC visits has increased, even though the Ministry of Health Strategic Plan target of 78% has been reached with 88.03% [49]. Study show that the number of ANC visits has a significant effect on TBM. However, implementing health services pregnant women must improve the quality of the services [49]. With the increase in maternal obesity found in this study from 2013 to 2018, it is highly recommended to detect early pregnancy at the first visit in the first trimester, when the high number of maternal BMI and nutrition status signals the start of TBM. Nonetheless, we recognize the limitations of analyzing ANC attendance data. Neither the frequency is correct nor the response can be determined from Riskesdas data.

This study found that, in 2013, children with working fathers had a reduced stunting risk. This may be because working fathers give more nutrition support to their children [50]. Fathers who have no or unstable employment may have more stress or unhappiness, making them unable to care for their child [51]. A study in Medan of unemployed fathers found that they had little involvement in giving attention to their children, especially in terms of fulfilling children’s nutrition, associated with unbalanced nutrition in their food [52]. Another variable that contributes to TBM in children is the number of household members. More household members are associated with lower odds of TBM. Other members can help the mother with nutrition intake or support her financially. With the joint effect of dependency and nucleation as an interaction term, indicate that in nucleated households with high dependency children have better health outcomes than in non-nucleated households with high dependency. Also in the case of weight-for-height, a positive effect is observed, reinforcing the argument that households that are nucleated and have high dependency tend to have better child health outcomes [53]. The household level is very important in causing malnutrition, such as anemia among children and obesity among mothers. Other studies have reported that this “household effect” most influences the risk factors in the child’s nutrition status. This study used MOR for the cluster level (household level) so the household highly influenced TBM status. It was reported that nuclear families have higher risk of anemia among children compared to non-nuclear families due to the limited resources, such as time and the number of family members paying attention to childrens’ diet [54]. In addition to that, mothers who live in a food insecure household also have greater risk of being overweight and obese since they adopt strategies that strive to protect their children to have more balanced and healthy diet. In food insecure household, mothers work hard to prevent hunger amongst their children, including prioritizing their children’s needs over their own. This may lead to overweight and obesity in mothers since they have fewer opportunities for recreational physical activity [55].

In 2018, the mother’s education influenced TBM status. The lower the mother’s education, the higher the risk of malnutrition in the child. Highly educated mothers can give their children various foods and know better how to serve them [56, 57]. A study in Bangladesh found that mothers with incomplete or no school education were 1.8 times more likely to have a stunted child than mothers who had completed primary school [58]. Combined data from 2013 and 2018 showed some similar factors that were linked with TBM. One such variable was the father’s education. The odds of a child with TBM increased with a non-academy-educated father. Highly educated fathers can help mothers with information about healthy foods and health facilities, which decreases TBM cases [50]. Null models obtained different MOR values between clusters, namely 1.37 in 2018 and 2.87 in 2013, according to the analysis. Higher MOR indicated a stronger cluster effect. As a consequence of the differences between the 2013 and 2018 research variation cluster, we predict that the likelihood of having TBM based on the risk factors will also differ.

According to our study, 24.9% of mother–child pairs had TBM. Most Latin American and South and Southeast Asian nations, including Guatemala, Colombia, Brazil, Malaysia, Indonesia, and Bangladesh, have studied maternal overweight and obesity, undernourishment in children, and their associated variables [24, 59]. However, the coexistence of TBM in mother–child pairs has not yet been researched. This study is the first to our knowledge to show the coexistence of overnutrition in mothers and undernutrition and anemia in children living in the same household. Our research has some limitations. First, a causal relationship between the explanatory and dependent variables could not be established. Second, information about dietary intake, physical activity level, health, and nutrition status during pregnancy were not available for the outcome measure of maternal overweight or obesity. Third, the mother’s nutritional status was examined only based on her BMI. When determining the type of overweight or obesity, BMI is less accurate than other techniques, including the waist-hip ratio, bioelectrical impedance technique, skinfold thickness, and Dual-energy X-ray absorptiometry. Despite these limitations, the use of a population-based, nationally representative sample was the study’s main strength. This study presented evidence that mother–child pairs living together may have an overweight or obese mother with a malnourished child and anemia, along with related variables. To prioritize nutrition intervention programs in Indonesia, policymakers might use the data as meaningful information.

## Conclusion

The coexistence of TBM in Indonesia among mother–child pairs is an alarming public health issue, despite the declining trend of undernutrition. Several identified risk factors among mothers, fathers, and children reflect that TBM is a life-cycle nutrition problem. Therefore, a life-cycle-based intervention approach from all relevant stakeholders starts from the family and community. This will play a critical role to prevent the increasing rate of TBM from the individual to the national level.

## Data Availability

The data that support the findings of this study are available from The Ministry of Health, Republic of Indonesia, under the Basic Health Research data, but restrictions apply to the availability of these data, which were used under license for the current study, and so are not publicly available. Data are however available with prior officially written permission of the Data Management Laboratory of NIHRD, Ministry of Health, Republic of Indonesia (datin.bkpk@kemkes.go.id).
